# A qualitative study of barriers and facilitators to pediatric early warning score (PEWS) implementation in a resource-limited setting

**DOI:** 10.3389/fped.2023.1127752

**Published:** 2023-03-15

**Authors:** Carolyn Reuland, Galen Shi, Mark Deatras, Mellinor Ang, Paula Pilar G. Evangelista, Nicole Shilkofski

**Affiliations:** ^1^ Johns Hopkins University School of Medicine, Baltimore, MD, United States; ^2^ Philippine Children’s Medical Center Department of Pediatric Critical Care, Quezon, Philippines; ^3^ Johns Hopkins University School of Medicine Department of Pediatrics, Baltimore, MD, United States

**Keywords:** limited resource, pediatric critical care, pews, early warning score, qualitative methodology

## Abstract

**Objectives:**

Globally, pediatric hospitals have implemented Pediatric Early Warning Scores (PEWS) to standardize escalation of care and improve detection of clinical deterioration in pediatric patients. This study aims to utilize qualitative methodology to understand barriers and facilitators of PEWS implementation at Philippine Children's Medical Center (PCMC), a tertiary care hospital in Manila, Philippines.

**Methods:**

Semi-structured interviews querying current processes for clinical monitoring, Pediatric Intensive Care Unit (PICU) transfer, and clinician attitudes towards PEWS implementation were audio recorded. In-person hospital observations served to triangulate interview findings. The Systems Engineering Initiative for Patient Safety (SEIPS) framework guided content coding of interviews to characterize work systems, processes, and outcomes related to patient monitoring and care escalation. Thematic coding was performed using Dedoose software. This model allowed identification of barriers and facilitators to PEWS implementation.

**Results:**

Barriers within PCMC workflow included: limited bed capacity, delay in referral, patient overflow, limited monitoring equipment, and high patient to staff ratio. Facilitators of PEWS implementation included support for PEWS adaptation and existence of systems for vital sign monitoring. Observations by study personnel confirmed validity of themes.

**Conclusion:**

Utilizing qualitative methodology to understand barriers and facilitators to PEWS in specific contexts can guide implementation at resource-limited hospitals.

## Introduction

Pediatric Early Warning Score (PEWS) systems are utilized in hospitals worldwide to delineate patient risk for clinical deterioration and standardize decisions about escalation of care. Scores are based on vital signs and clinical data, including heart rate, respiratory rate, blood pressure, neurologic status, respiratory effort, and temperature. These data help predict patient deterioration and guide resulting course of action. Early warning scores have been shown to be accurate predictors of need for ICU level care, and better predictors of clinical deterioration than physician opinion alone (1–9). Some implementation studies have demonstrated improved clinical outcomes as a result of early recognition of patient decline, allowing for earlier intervention (9).

It is important to modify PEWS systems to fit specific hospital contexts worldwide, particularly in limited-resource settings (i.e., centers with high patient-to-staff ratios, limited monitoring and treatment equipment, etc.) (1–3, 5, 10). PEWS scores in highly-resourced hospitals rely on presence of telemetry, while limited-resource settings rely on clinical measurements available without technology, i.e., manually measured heart rate and capillary refill. These scores are used to guide decisions about invoking assistance from rapid response teams (RRT), groups of clinicians who urgently present to the patient's bedside for evaluation and intervention. In settings lacking formal RRT programs, scores can be used as a guide for closer observation or referral to higher level of care.

The Philippine Children's Medical Center (PCMC) is a 200-bed tertiary care pediatric referral center in Manila, Philippines. A government-funded institution and teaching hospital, PCMC is often overburdened with large volumes of high acuity patients, frequently exceeding bed capacity. Monitoring equipment is typically available only in the pediatric ICU (PICU). PCMC currently does not utilize scoring systems for making escalation-of-care decisions, and overflow of severely ill patients in the emergency department (ED) causes distress to patients, families, and staff, and may be a source of worsened patient outcomes. The goal of this study was to characterize clinical monitoring systems and care escalation at PCMC to understand how to modify a PEWS system to be context- specific. The study aims to first understand how escalation of care is managed within a resource-limited setting, and second, to evaluate how the system may provide barriers and facilitators to implementation and adaptation of a PEWS system.

This study also aims to involve PCMC clinical staff in assessing feasibility of a warning score system prior to its implementation, a goal derived from normalization process theory, which postulates that inclusion of stakeholders in implementation processes improves integration of new programs into workflow. Normalization process theory provides a rationale for this type of pre-implementation study: to optimize conditions for successful adaptation of new workplace tools such as PEWS, hospital staff should be involved in evaluating tool feasibility. By analyzing existing work structures at PCMC, potential barriers and facilitators to future PEWS implementation were identified. Findings from the study may be used to guide adapted PEWS programs to optimize fit within PCMC contexts.

## Materials and methods

### Study setting and participant recruitment

The study was approved by institutional review boards at both Johns Hopkins School of Medicine and Philippine Children's Medical Center. The study employed qualitative methodology to collect information about PCMC workflow. Twenty-four semi-structured interviews were conducted in English by interviewers external to the PCMC system, with six nurses, six residents, six fellows, and six attending physicians at PCMC who gave written consent prior to interviews. While there are occasionally other individuals involved in patient care, such as respiratory therapists and parents, we chose to include those who would be most integral to a PEWS implementation process, and who did not require the use of a translator. Participants were a purposive sample, recruited from PCMC staff by study investigators based on availability and willingness to participate. Amongst the staff approached, there were no refusals to participate. Interviewees were informed that answers would serve to characterize PCMC workflow and understand if a PEWS system may have potential benefit at PCMC. Specific barriers and facilitators to implementation were evaluated based upon in-person observations and semi-structured interviews with clinical staff. The interviews were thematically coded using the Systems Engineering Initiative for Patient Safety (SEIPS) framework, allowing for systematic understanding and organization of information collected during interviews.

### Qualitative interviews and in- person observations

In keeping with qualitative methodology and an ethnographic approach, interviews were conducted until thematic saturation was reached by two study personnel in private locations at PCMC (offices and conference rooms), typically lasting 10 to 15 min. Semi-structured interview questions focused on processes of patient monitoring and escalation of care at PCMC (see Supplementary Materials Appendix S1). Two study personnel each spent one week shadowing providers on rounds and recording interprofessional interactions as field notes in the ED, PICU and pediatric wards, observing processes of patient evaluation, provider communication, and patient transfer. Salient observations were recorded by hand as field notes during rounds. Common themes from observations and field notes supported data garnered from interviews, and served to triangulate interview findings and increase validity of outcomes through convergence of information from different sources.

Each interview was audio recorded, de-identified and then transcribed by GoTranscript. Transcripts were analyzed by two independent reviewers using Dedoose coding software. The SEIPS framework was used to organize the coding process.

### SEIPS framework

**Figure 1:**
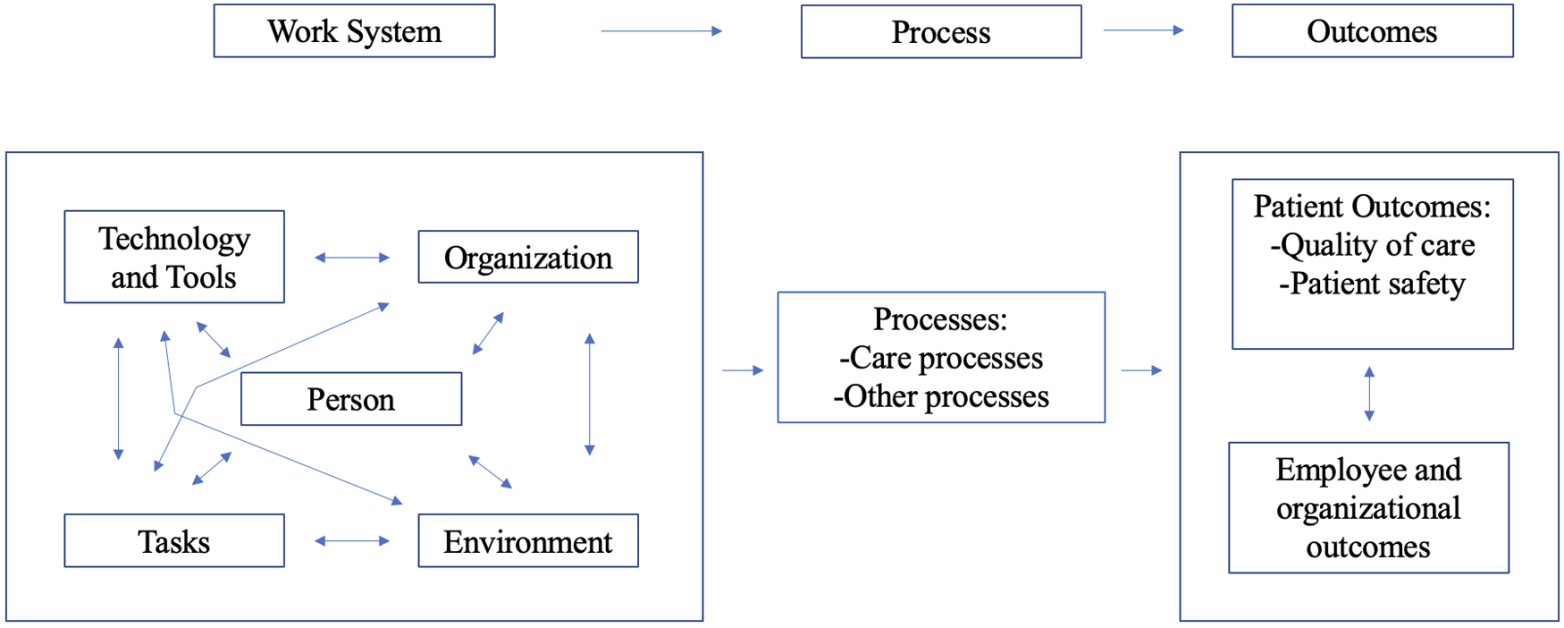
Systems engineering initiative for patient safety (SEIPS) model (11). (This diagram is an adaptation of the published version).

The SEIPS framework was chosen as a method of organizing information derived from interviews, allowing for thematic organization of various aspects of PCMC workflow. It allowed for understanding of various factors at play in the system that contribute to issues with escalation of care at PCMC, and how a new tool might fit into this system to address these issues. Within the framework, each heading allows for exploration of different aspects of the system. The “technology and tools” segment discusses availability and utilization of materials and resources for functionality of the system as a whole. The “person” heading allows for exploration of important “actors” in the system. The “tasks” heading highlights specific jobs to be accomplished within the work system. The “organization” heading allows for understanding of the institution in which these actors operate. The “environment” section explores context and setting in which the organization operates. The arrows on the SEIPS graphic indicate complex interactions between each of these sectors of a work system (see Figure 1). These headings of the SEIPS framework served as parent codes within Dedoose coding software. Two study personnel read transcripts and assigned statements to these parent codes, and to child codes within these as more specific themes were identified. Themes from in-person observations were also recorded and sorted into this coding system. Study personnel then compared codes and organized findings into a final presentation of PCMC's work structure.

## Results

Results from interviews and observations can be divided into two categories: (1) findings about existing work structure with regard to clinical monitoring and escalation of care, and (2) specific barriers and facilitators to implementation of PEWS. Results were derived from interview statements and triangulated by direct observations of workflow processes by study personnel, in keeping with qualitative methodology. While there is significant overlap between findings from each of these categories, it is important to separate them based upon two overarching goals being analyzed within the SEIPS framework: (1) effective escalation of care and (2) successful implementation of a PEWS system. In keeping with “thick description” processes in reporting qualitative study results, we have utilized direct quotations from interviews and observations as examples within several categories (see Table 1).

**Table 1:** Exemplar thick description quotations from key informants during qualitative interviews.

Quote	Key Informant	Parent Code and Theme
“*They need to bag, which is typical for the parents.”*	Pediatric Resident, 27, Female	Person – parental involvement
“*You will first interview downstairs in the ER, of course, and then we will order the labs and then we refer to the PICU fellow and the PICU resident. Then, we will wait for their answer. After around 10-15 min, they will go down already to see the patient.”*	Pediatric Resident, 28, Female	Organization – PICU team as rapid responder
“*The tendency is they stay long in the emergency room because we don’t have available beds … They usually get health-care associated infections.”*	Pediatric Resident, 29, Female	Environment – overcrowding

Existing PCMC Work Structure is elucidated in each category below:

*Technology and Tools*: PCMC operates without electronic medical records; all patient charting is performed on paper. Cardiac and respiratory vital sign monitoring equipment is not available, except in the PICU. Insufficient quantities of blood pressure cuffs or properly sized cuffs for smaller patients was mentioned frequently in interviews. Lack of ventilators to treat patients with respiratory failure was noted in observations and by many interviewees. Another aspect of technology noted was utilization of cellular telephones for communication about patient condition. The cellular signal in the hospital is often sporadic, according to many interviewees. Finally, the hospital's patient transport elevator was non-functional at the time of the study. All transfers between floor were performed on ramps, which was time-consuming and reliant on personnel availability.
*Person*: Care teams at PCMC are composed of nurses, residents, fellows, attending physicians, respiratory technicians, and at times, patients' parents. Importantly, the ratio of providers to patients are diffusely low across all areas of the hospital. As a result of this mismatch and lack of sufficient clinical staff, parents are often directly involved in the care of their children. For example, it was frequently observed and mentioned in interviews that parents, rather than trained clinical personnel, may be responsible for manually ventilating their children using bag-valve masks attached to endotracheal tubes for days at a time until a ventilator is available.
*Tasks*: Major tasks that emerged in discussions with staff were vital sign collection, patient stabilization, and patient transfer.
*Organization*: PCMC is a teaching hospital. Residents completing three-year pediatric training programs each have specific roles in patient care. Nurses are assigned to locations throughout the hospital and are responsible for collecting vital signs, administering medications, and other health maintenance tasks. When a patient appears to be unstable, a nurse or first year resident may refer to a senior resident or directly to PICU fellows to discuss transfer.
*Environment*: PCMC is located in Manila, Philippines. It is the largest pediatric referral center in the country, receiving large volumes of high acuity patients with a high case mix index. Particularly during summer months, seasonal prevalence of endemic infectious diseases such as dengue hemorrhagic fever and leptospirosis leads to a rise in acutely ill patients presenting to the ED. The hospital is government-owned and therefore treats all patients, even those who cannot afford payment. As the hospital cannot turn down patients due to lack of financial resources, it is often overflowing with patients who cannot afford better- resourced private hospitals.

### Barriers and Facilitators to PEWS Implementation

These same thematic categories from the SEIPS framework may be used to organize observations and interview comments about barriers and facilitators to PEWS implementation.

*Technology and tools*: A barrier to PEWS implementation is lack of tools necessary for collection of certain vital signs, including cardiorespiratory monitoring and blood pressure equipment. Additionally, if PEWS scores were able to be calculated, communication of scores to other care team members depends upon the hospital's unreliable cellular signal. Finally, even if a PEWS score necessitated PICU transfer, lack of elevators is another barrier to expeditious transfer. However, an important facilitator to PEWS implementation is that many clinical signs scored in PEWS are collected manually by nurses and do not necessitate technology.
*Person*: A major barrier to PEWS implementation is patient overflow and relatively low number of clinicians. If providers lack time for an extra step in patient management due to high patient load, a PEWS system would likely not be accepted or useful. However, one important facilitator discovered during interviews was that attitudes toward potential PEWS implementation was uniformly positive across all interviewees.
*Tasks*: A barrier to PEWS implementation is that existing provider workload is extraordinarily high, resulting in little time for extra monitoring steps. However, one facilitator to PEWS implementation is that the vital signs that make up PEWS are already routinely collected. This suggests that the addition burden of calculating PEWS scores within the list of tasks for nurses or residents may not extend beyond existing duties.
*Organization*: One barrier to PEWS implementation within the organization of PCMC is that the ED functions like a ward due to limited bed space, often housing patients for days until inpatient beds are available. Most hospitals that use PEWS systems have implemented scores on standard wards and not as triage measures in ED settings (5). Even if PEWS were utilized in the ED and dictated that patient transfer was required, lack of bed space may preclude transfer. Importantly, a facilitator to implementation is that the organization has an existing chain of command that utilizes PICU fellows and residents as a de facto RRT for patients requiring acute care. Thus, PEWS could be utilized to dictate not only need for physical transfer to the PICU, but a need for closer monitoring from the PICU team, an existing system in the PCMC organization.
*Environment*: The overcrowding at PCMC might prevent PEWS systems from being utilized by busy clinical staff. Additionally, even if a PEWS score indicated need for PICU care, lack of bed vacancy could preclude transfer, thus reducing efficacy of the PEWS system in moving patients to hospital areas most appropriate for their condition. However, it may also be feasible to utilize PEWS to guide initiation of ICU-level care outside the ICU itself (involving more frequent vital sign monitoring or other ICU-level interventions that could be performed at the bedside). Additionally, one facilitator to PEWS implementation is the teaching structure of PCMC and the reported desire to standardize algorithms and decision-making processes amongst more junior clinicians.

## Discussion

Decisions about escalation of care in limited-resource hospitals are often difficult, unstandardized, and a cause of concern to hospital staff (12, 13). The usage of early warning score systems in resource-limited environments poses a dilemma: does addition of new tasks to a clinician's already extensive workload outweigh benefit of information given by the score? Most studies employing these systems in low-resource settings have found that modification of the score to best fit the needs of staff in each context has improved outcomes and adoption of the practice (1, 12, 14, 15). Thus, a program like this should be implemented only once there is broad understanding of the setting and its actors.

The qualitative methodology employed in this study allowed for comprehensive understanding of the PCMC workflow. Though many studies have evaluated outcomes of early warning scores, few have formally studied feasibility of tool implementation within the site's existing workflow (1–5, 7, 9, 10, 16). Evaluating the workflow of PCMC within the SEIPS framework allows for thorough understanding of factors that will affect implementation and adoption of a PEWS system. This model was developed with the goal of ensuring maximal patient safety by considering multiple aspects of a work system. It was recently described as the framework used to assess barriers and facilitators to implementation of Integrated Management of Childhood Illness (IMCI) guidelines and Emergency Triage Assessment and Treatment (ETAT) program at a hospital in Malawi (17).

Reports detailing implementation of PEWS at other hospitals have illustrated findings similar to those in the present study. One study at a hospital in Guatemala attributed success of PEWS implementation to nursing buy-in, institutional dedication to quality improvement, and open communication throughout implementation (2). This finding suggests that positive attitudes of nurses and clinicians interviewed at PCMC could serve as facilitators to PEWS implementation. Additionally, validation of a score in a similarly low-resourced hospital in Rwanda was supported by simplicity of the tool, which avoided blood pressure measurements as well as use of more subjective assessments such as work of breathing (10). This score simplification allows for data collection by healthcare workers with less training. While lack of sufficient training of personnel at PCMC did not surface as a barrier to PEWS implementation, simplification of the tool to be short in length was a suggestion raised by a PICU fellow.

To our knowledge, this study is the first of its kind to utilize normalization process theory as part of its rationale. This theory suggests that by involving stakeholders in the action of performing a feasibility study, adoption, buy-in and understanding of the tool are improved (18). It is defined by four principles: coherence, cognitive participation, collective action, and reflexive monitoring (19). This study focuses on the first two: coherence (functionality of a system given practical constraints of a certain context), and cognitive participation (involvement of clinicians in the development process). Other studies have examined effects of modifying PEWS systems in a limited resource setting on its efficacy but have not studied the role of involving clinicians who will use the study in its development (5, 10, 20).

Even in well-resourced settings, adherence to newly implemented PEWS systems is variable, and often a barrier to functionality of these systems (21). Utilizing principles of normalization process theory allows for better chance of adherence to new practices: clinicians who have helped guide tool development to best fit their existing workflow are more likely to utilize it (19, 22). This study sets the stage for future implementation and hospital policy development of a PEWS system.

This study is novel in its use of the SEIPS framework for organization and coding of interview findings in a feasibility evaluation of PEWS. SEIPS has previously been used to guide development of patient safety interventions (23). To our knowledge, this study is the first to employ this framework in the evaluation of a PEWS system in a limited resource hospital.

One limitation of the study is that, although the interviews were conducted with fluently English-speaking participants, many participants' first language is Tagalog. Thus, it is possible that some interview responses could be limited by linguistic contextualization and individual comfort with English. Another limitation is the degree of study generalizability, given that it was conducted at a single institution. However, this may be tempered by potential applicability of findings to hospitals in other countries that are similarly resourced and structured.

## Conclusions

Despite many challenges within the work system, the PEWS system may be feasible to integrate into limited-resource settings in the presence of existing structures within the organization and perceived acceptability by hospital stakeholders who would be involved in implementation. Modified PEWS scoring can be used in limited resource contexts to standardize decisions about consultation of higher-level care teams. Modification of PEWS systems to fit specific hospital contexts can be achieved through evaluation of hospital work systems within a patient safety framework. Involvement of stakeholders in the adaptation of PEWS systems for specific hospital contexts may improve adherence and efficacy of these systems.

## Data Availability

The raw data supporting the conclusions of this article will be made available by the authors, without undue reservation.
