# Supplementary material for: A qualitative study of barriers and facilitators to pediatric early warning score (PEWS) implementation in a resource-limited setting
Source: Front Pediatr. 2023 Mar 15;11:1127752. doi: 10.3389/fped.2023.1127752 (PMC10050749; doi:10.3389/fped.2023.1127752)
Supplement: Supplementary file 1 [file Table1.docx]

Appendix 1 – Semi Structured Interview Question Guide

**Questions for Nurses:**

- What is your title and experience level/how long have you been a nurse?
- What is your satisfaction with the patient transfer/endorsement process with nurses from other areas of the hospital?
- What is the nurse-to-patient ratio in your area of work?
- What terminology do you use to quantify severity of a patient’s condition?
- Do you collect vital signs, and if so, which ones? How frequently? Are there barriers to collection?
- What do you look for to determine if a patient needs escalation of care?
- Who do you communicate with when you determine escalation of care is needed? How does this process go? What are barriers to communication with others?
- What are barriers to transfer of patients to PICU?
- What are your attitudes about the possibility of implementing a PEWS system?

**Questions for Physicians (Residents/Fellows/Consultant Attendings):**

- What is your title and experience level/how long have you been a doctor?
- What is your role in patient care and patient transfer?
- What is your satisfaction with the endorsement process with doctors from other areas of the hospital?
- What is the nurse-to-patient ratio in your area of work? Resident to patient ratio?
- What terminology do you use to quantify severity of a patient’s condition?
- Do you collect vital signs, and if so, which ones? How frequently? Are there barriers to collection?
- What do you look for to determine if a patient needs an escalation of care?
- Who do you communicate with when you determine escalation of care is needed? How does this process go? What are barriers to communication with others?
- What are barriers to transfer of patients to PICU?

What are your attitudes about the possibility of implementing a PEWS system at PCMC?
